# Associative Learning in a Conspiratorial Frame

**DOI:** 10.1177/17470218251396472

**Published:** 2025-11-01

**Authors:** Tom Kelly, Michael Hattersley, Elliot A. Ludvig

**Affiliations:** 1 Department of Psychology, University of Warwick, Coventry, UK; 2 Sutherland School of Law, University College Dublin, Ireland; 3 Slovak Academy of Sciences, Institute of Experimental Psychology, Bratislava, Slovakia

**Keywords:** conspiracy theories, associative learning, belief updating, blocking

## Abstract

Conspiracy beliefs are widespread and can lead to deleterious societal outcomes, yet little research has examined how they are learned. In associative learning, blocking occurs when people learn less about a novel cue in the presence of another causally predictive cue. Here, blocking was examined in a conspiratorial context. In the task, participants were told about a foreign politician and a possible conspiracy: This politician had allegedly been poisoned at a given location. Participants were then presented with a set of pairings between locations and illness; the conspiracy-congruent location was always presented together with a novel location. Learning about this novel location was blocked by the conspiracy-congruent location and, on aggregate, participants endorsed the conspiracy theory. There was, however, no evidence that conspiracy theorists were more likely to demonstrate blocking in general. Conspiracies can thus be acquired and maintained through similar associative-learning processes as other beliefs.

## Introduction

Endorsement of conspiracy theories is a widespread, growing phenomenon that has been linked with troubling societal outcomes (Jane & Flemming, 2014; Jolley & Douglas, 2014a, 2014b; Lewandowsky et al., 2013; van der Linden, 2015). Despite significant research into the individual differences that predispose individuals to endorse conspiracy theories, there has been little research on the mechanisms which underpin the learning or maintenance of such beliefs. The principles of associative learning have previously been used to understand high-level concepts as diverse as political beliefs and superstitions (Blanco et al., 2015, 2018; Fernandez & Timberlake, 2020; Skinner, 1948; Staddon & Simmelhag, 1971). This paper adapts a classic associative learning paradigm, blocking (Dickinson et al., 1984; Kamin, 1969), to examine the adoption and maintenance of a new conspiracy theory. If, as in other beliefs, blocking plays a role, then prior exposure to a conspiratorial explanation should prevent learning about alternative explanations.

Previous work suggests that conspiracy believers process information differently. Conspiracy believers jump to conclusions (Hattersley et al., 2022; Moulding et al., 2016; Pytlik et al., 2020), think intuitively (Hattersley et al., 2022; Swami et al., 2014; van Prooijen, 2016), and promote narratives consistent with confirmation bias (Quattrociocchi et al., 2016). Such studies, however, employ correlations between specific processing variables and an aggregate measure of conspiracy belief: lists of specific conspiracy theories or more general statements regarding a conspiracy mentality (Brotherton et al., 2013; Swami et al., 2010, 2011). As such, these measures do not consider how participants react to a novel conspiracy theory.

A handful of studies have employed novel conspiracy theories (Bost & Prunier, 2013; Imhoff & Lamberty, 2017; Swami et al., 2011; van Prooijen & van Dijk, 2014). For instance, van Prooijen and van Dijk (2014) provided a fictitious account of an opposition leader from Benin who was either killed or injured in a car accident, finding that major causes (i.e. conspiracies) are linked to major consequences (i.e. death). The focus of these studies, however, has not been the mechanisms of belief learning or maintenance, reflecting the lack of process-driven investigations into conspiracy learning.

This paper presents an experimental, process-driven investigation into the learning and maintenance of a novel conspiracy belief. The experiment employs a well-established associative-learning effect: blocking (Kamin, 1969). In blocking, a previously learnt stimulus–outcome pairing interferes with or blocks future learning, a behaviour regularly exhibited in humans (Dickinson et al., 1984). Specifically, once a given stimulus is known to predict an outcome, this blocking stimulus can reduce learning about a novel stimulus and this outcome, provided that this second, blocked, stimulus is exclusively observed alongside the blocking stimulus. The blocking effect has been demonstrated in a variety of different tasks, including fear conditioning, eye-blink conditioning and causal learning (Lovibond et al., 2003; Martin & Levey, 1991; Mitchell & Lovibond, 2002).

Within the associative canon, blocking is a promising candidate for providing insight into the learning of conspiracy beliefs. Blocking is a foundational effect with widespread applicability (Maes et al., 2016; 2018; Soto 2018), and the order of learning in blocking maps onto specific “breaking news” scenarios where individuals may encounter a conspiracy theory before the full facts have been established. In addition, as people typically encounter conspiracies through the descriptions of others, studying conspiracy learning requires an approach that supports the incorporation of explicit descriptions, as has been shown in blocking (Kelly & Ludvig, 2025; Le Pelley et al., 2005; Shanks, 1991). This information format reflects the fact that most people who endorse conspiracy theories have not personally observed the events about which they have strong views, but rather have learned about those events from others (e.g. the moon landing or 9/11). Indeed, there is even evidence of hostility toward genuine witnesses of alleged conspiracy scenarios, if those witnesses do not comply with conspiracy theorists’ views (Ronson, 2011).

To that end, this experiment adapted an allergy-detection paradigm common in the blocking literature to a conspiratorial frame (Lovibond et al., 2003; Spicer et al., 2021; Van Hamme & Wasserman, 1994). In the conspiracy-learning task, participants were asked to identify which places their patient visited on a given day made (or did not make) them ill. First, participants learned which locations cause illness. Following this initial training, participants were then introduced to a fictional politician from Benin and explicitly informed that a certain location causes him to be ill. The information reported was congruent with a possible governmental conspiracy, without explicitly stating that a conspiracy had occurred. The experiment sought to establish if this conspiracy-congruent information about one location blocks learning about other locations that could also have induced illness. Following this experimental task, participants completed psychometric measures to gauge the relationship of existing conspiratorial beliefs to behaviour in the blocking task.

This paper primarily investigates a *Conspiracy Blocking* hypothesis: An explicit conspiratorial description of a cue–outcome relationship will produce a blocking effect and prevent learning about the location–illness relationship for other paired locations. Additionally, this paper will consider a secondary *Conspiracism Correlation* hypothesis: Across individuals, the degree of blocking will correlate with their self-reported degree of general conspiratorial belief. The hypotheses, design, and analysis plan were pre-registered: (https://osf.io/m3ywf/overview). All methods and analyses followed the pre-registered plan unless otherwise indicated.^1^ All materials, data and analysis scripts are available at the same link.

## Method

### Participants

Two hundred seventy-six participants were recruited from the Prolific Academic online recruitment system (Prolific.co). Participants (157 male, 118 female, 1 unknown) were paid a £2 participation fee plus a performance bonus of up to £3 (mean bonus = £1.91). Participants were aged between 18 and 75 with an average age of 29.1 years. Participants were screened to ensure they were fluent in English and that they were using a computer rather than a phone or a tablet using Prolific’s filters. Participants were first linked to Qualtrics, where they were presented with an information sheet and consent form. Participants who did not provide consent were redirected back to the Prolific website. The online experimental conspiracy-learning task was completed on Pavlovia.org. Following task completion, participants were redirected back to Qualtrics to fill in the psychometric questionnaires and for a debrief before returning to Prolific. Participants were provided with a password at the end of the experimental task that encoded the bonus (if any) to be paid for their performance.

The experiment used a within-participant experimental design with additional correlational measures. Following Schönbrodt and Perugini’s (2013) recommendation that 250 participants are required for a stable estimate in correlations for typical psychological variables, we gathered a sample of 276 participants (250, plus a 25-participant buffer for incomplete responses and one extra collected by Prolific to replace a timed-out participant). Power analysis using G*Power (Faul et al., 2007, 2009) shows that a sample of 250 gives 99% power to find a correlation of .2 with an alpha of .01. For this design, with 90% power and an alpha of .01, 275 participants could detect an effect size as small as *d* = 0.23. Ethical approval was received from the University of Warwick Psychology Department Ethics Panel.

As pre-registered, all incomplete files were excluded from the analysis. Two participants completed the conspiracy-learning task having either exited or crashed during their first attempt; their completed files were excluded. Two participants who completed the task twice had their second datasets removed. Two participants for whom the program crashed during the study were paid for their partial completion but excluded. These exclusions resulted in a total of 270 participants who completed the conspiracy-learning task. One participant’s data were not saved for the conspiracy-endorsement measure at the end of the experiment, but because they had completed the experimental task, their data were retained.

Two hundred sixty-five participants completed at least some of the questionnaire measures following this task. Two hundred thirty-one participants completed every item in the General Conspiracy Belief Scale – GCBS (Brotherton et al., 2013), and 212 participants completed every item of the Belief in Conspiracy Theories Inventory – BCTI (Swami et al., 2010, 2011). Any participant who did not complete a questionnaire had all their responses for that measure removed, although their responses on other measures were retained. A few participants (three for the CGBS and four for the BCTI) completed all measures within one or more of the questionnaires despite having quit, crashed, or otherwise prematurely terminated their participation in the conspiracy-learning task. Additional information regarding the Social and Economic Conservatism scale (Everett, 2013) and the self-rated left/right scale is provided in the supplementary materials.

### Stimuli and Materials

Stimuli consisted of 10 images representing locations (see Supplemental Material). The first three images, representing a coffee shop, church and supermarket, comprised their own set of three and only occurred in Phase 1a. This limitation was included to prevent confusion (going for coffee and a restaurant are similar) and to have similarly plausible causes in Phase 2 (i.e. supermarkets and churches are almost exclusively public environments, whereas the other seven can be more private, smaller spaces). The remaining seven locations could play any role within the task. Each compound stimulus was shown with each location on the left or right of the screen equally often. The experimental roles of the specific stimuli in the task were randomised between participants. The two conspiracy-belief questionnaires, the Generic Conspiracist Belief Scale (Brotherton et al., 2013) and the Belief in Conspiracy Theories Inventory (Swami et al., 2010, 2011), and two measures of political orientation, the Social and Economic Conservatism Scale (Everett, 2013) and a self-rated, relative-rank measure of left-right wing preference, were presented with Qualtrics.

### Procedure

After providing informed consent, participants were informed that their role in the experiment was that of an allergist and their task was establishing which locations caused illness. Participants were told they would see locations where their patient went on a given day. Their task was to establish if their patient was ill that evening. Participants were introduced to the scale used, the incentive structure, and the feedback. After 100 ms, participants could press the space bar to proceed past the instructions. Participants were asked not to make any notes. The experiment was created with PsychoPy version 2020.1.3 (Peirce, 2007, 2009).

The experiment consisted of four phases. Table 1 details the full experimental design. Phase 1a (Pre-training) consisted of 45 trials (30 compound, 15 elemental), where participants were trained that some stimuli caused illness. Table 1 shows how, during the pre-training, participants were presented with compound stimuli consisting of two locations. This training phase served to familiarise participants with the task and allowed them to experience how causation operated in this environment. Previous work with a similar task has shown that this pre-training is important for the appearance of blocking with abstract shapes (Kelly & Ludvig, 2025). This phase was immediately followed by Phase 1b (Description). In Phase 1b, the *Described Blocking Stimulus* was introduced, where participants were explicitly informed that a specific location causes illness. In Phase 2 (Blocking), for 30 trials, participants were again presented with pairs of stimuli which caused or did not cause illness. In Phase 3 (Single Stimuli), participants were presented with 35 trials with individual locations to assess what people learned about each location. Thirty of these trials presented familiar location stimuli, and five presented a novel location stimulus. In Phase 4, across 40 trials, a subset of stimulus pairs was used for a two-alternative forced-choice (2AFC) task.

**Table 1.** Experiment Design. Each Column Details the Stimuli in a Given Phase.

Phase 1a: Pre-training	Phase 1b: Description	Phase 2: Blocking	Phase 3: Single stimuli	Phase 4: 2AFC
XY+ XZ+ YZ− X+ Y− Z−	A = illness	AB+ CD+ EF−	All individual stimuli (includes novel stimulus N)	All stimulus combinations except D and F
Training set			No Feedback No training set	No feedback No training set

*Note.* 2AFC = two-alternative forced-choice.

The key stimuli are the *Described Blocking Stimulus* (A), the *Described Blocked Stimulus* (B) and the *Reinforced Controls* (C and D). Table 2 summarises all the stimuli in the experiment. Participants were first told the Described Blocking Stimulus was linked with illness in Phase 1b. This stimulus was then paired with the Described Blocked Stimulus in Phase 2. The Reinforced Controls have equivalent histories of reinforcement to a Blocked Stimulus and were introduced at the same time. They were not, however, paired with a Blocking Stimulus (i.e. a stimulus with a prior association with illness). Therefore, if participants learn differently about a Blocked Stimulus and a Reinforced Control, the reason is due to prior learning about the Blocking Stimulus blocking learning about the Blocked Stimulus.

**Table 2.** Stimuli and Their Characteristics.

Label	Stimulus	Introduced	P2 pair	P2 reward
Described blocking stimulus	A	P1b	B	Y
Described blocked stimulus	B	P2	A	Y
Reinforced control	C	P2	D	Y
Reinforced control	D	P2	C	Y
P2 unreinforced	E	P2	F	N
P2 unreinforced	F	P2	E	N
Novel control	N	P3	N/A	N/A
Training reinforced	X	P1a	N/A	N/A
Training unreinforced	Y	P1a	N/A	N/A
Training unreinforced	Z	P1a	N/A	N/A

*Note.* P = Phase.

**Figure 1.**
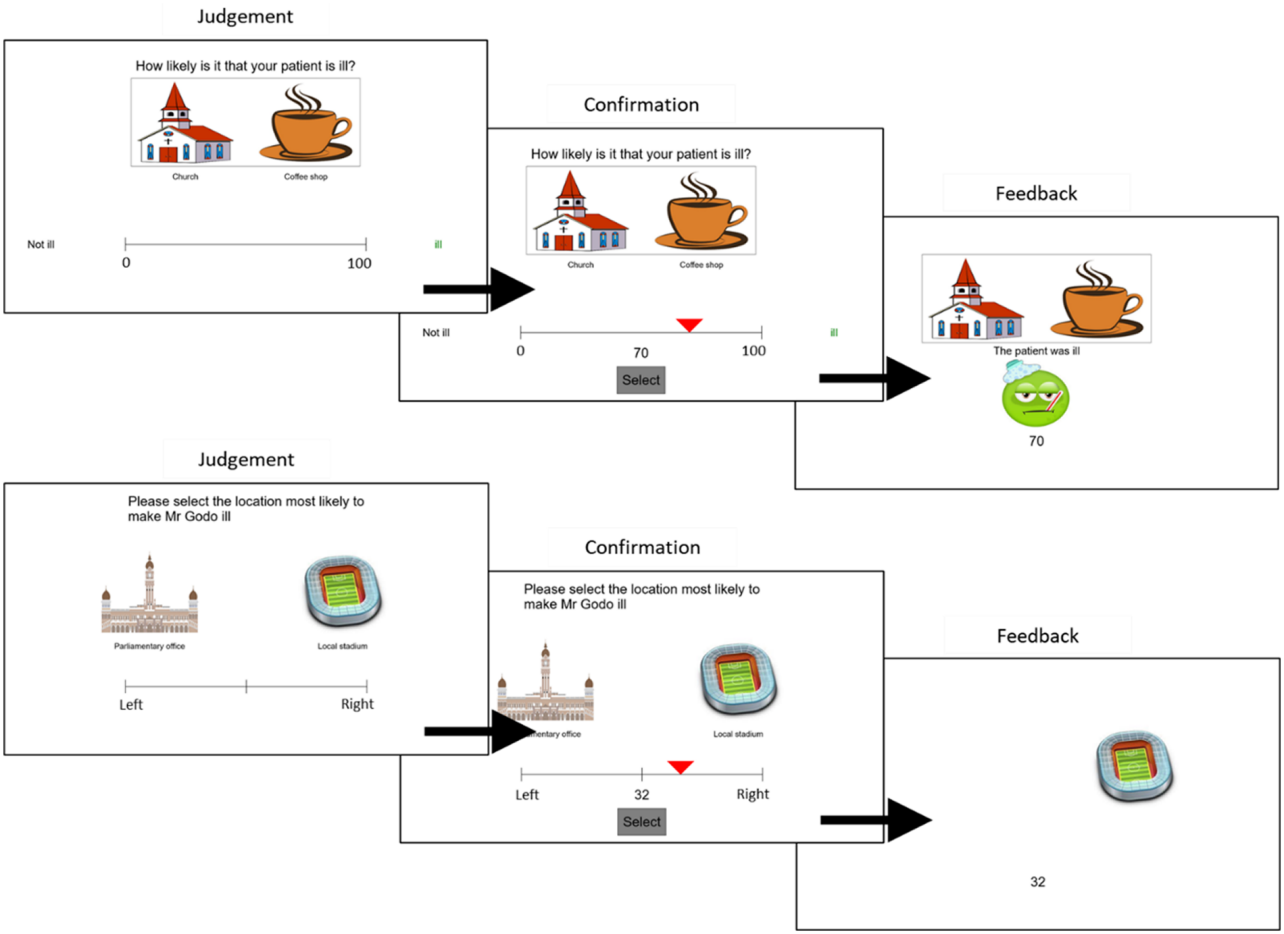
Top panels. Schematic of Phase 1a/2 trial. The first (top-left) panel shows a screenshot of the main judgement task where people were presented with two locations and asked to judge on a scale of 0-to-100 how likely the patient (Phase 1a) or Mr Godo (Phase 2) was ill. The second panel shows the participants’ initial response of “70” prior to confirmation. The third panel shows a screenshot of the feedback screen, after the participant selected “70,” and the location did indeed cause illness. Bottom Panels. Schematic of a Phase 4 two-alternative forced-choice trial. The first (bottom-left) panel shows a screenshot of the judgement task where people were presented with two locations and asked which stimulus was more likely to make Mr Godo ill. The second panel shows the participant’s initial response of “32” on the stadium side of the scale prior to confirmation. The third panel shows a screenshot of the feedback screen where participants received visual confirmation of their choice.

During each trial in Phases 1 to 3 (except in Phase 1b: Description), participants saw a 0-to-100 scale at the bottom of the screen as they were presented with the stimulus. In Phase 1a, Participants were asked “How likely is it that your patient is ill?” In Phases 2 to 3, this question became “How likely is it that Mr Godo is ill?” (see below). Participants responded by selecting the appropriate location on the scale with their mouse and by confirming their choice using the “select” button that appeared once they made an initial choice. Participants could change their answer between making an initial selection and pressing select. See Figure 1 (top panels) for a schematic of a Phase 1a/2 trial.

In Phase 1b (Description), participants were introduced to the story of their patient “Mr Godo.” The story of Mr Godo was directly inspired from the conspiracy-theory literature (van Prooijen & van Dijk, 2014). Participants were told that Mr Godo was a prominent opposition MP in Benin who possibly had enemies and that his previously existing allergies have been recently getting worse. Full text is provided in the Supplemental Material. Participants were able to advance from the page containing Mr Godo’s story after 7 s.

This introductory information was then followed by news reports stating that a specific location is the cause of Mr Godo’s illness and that this location is owned/controlled and/or managed by supporters of the governing party. The precise wording of this message changed depending on exactly how the stimuli were initially randomised, so that the wording fit the location. Participants were again able to advance from this page after 7 s. Whilst participants could infer from this description that a conspiracy had occurred, at no point was this made explicit. Following this description of the news report, the location stimulus corresponding to this information (e.g. a picture representing a gym) and a short message indicating this location (e.g. the gym) causes illness appeared for 5 s.

In Phases 1a and 2, after each response, participants received feedback about their choices for 1.5 s. The feedback consisted of several elements: The scale and the select button vanished, plus written text and an image appeared indicating whether the stimulus caused illness. Immediately following this 1.5-s feedback period, the next trial began. In Phase 3, the feedback lasted for 1 s and only indicated what number was chosen but gave no information about correctness or illness. Interim aggregate feedback (total bonus points) was provided for 3 s at the end of Phases 1a and 2.

Phase 4 was a 2AFC task. See Figure 1 (bottom panels) for a schematic of a Phase 4 trial. Participants were asked to select the location most likely to make Mr Godo ill using a 100-to-0-to-100 scale at the bottom of the screen as they were presented with the stimuli. The first 100 meant the left option was more likely, and the second 100 represented the right option being more likely. Participants responded by selecting the appropriate place on the scale with their mouse and confirming their choice using the “select” button that appeared once they made an initial choice. There were 10 pair-wise comparisons in total: A versus B, A versus C, A versus E, A versus N, B versus C, B versus E, B versus N, C versus E, C versus N and E versus N (see Table 1). The two omitted stimuli (D and F) were excluded to reduce the total number of trials, as these stimuli already had a functionally equivalent stimulus tested (C and E). Participants received visual confirmation of their choice for 1 s following their decision. If participants selected the value 0, both stimuli remained on the screen for 1 s as feedback.

Following completion of the experimental task, participants were asked to use the same 100-to-0-to-100 scale to answer the No-to-Yes question: “Did the Benin Government poison Mr Godo?” This question was used as a measure of conspiracy endorsement. Participants were informed that this question was not incentivised prior to answering. Participants were then informed of their final bonus and payment before being directed back to Qualtrics to complete the questionnaires and finish the study.

#### Incentive Structure

The experiment was fully incentive compatible. In brief, for Phases 1 to 3, if participants rated a causal stimulus above 0, they gained the corresponding number of points, and they similarly lost points for rating a non-causal stimulus above 0. In the 2AFC task, participants earned the corresponding number of points for choosing a stimulus that was more likely to be causal and lost points for choosing a stimulus less likely to be causal. Participants earned £1 for each 3,000 bonus points earned. There was a maximum of 9,000 bonus points available in the experiment (which would provide a £3 bonus). The bonus was rounded to the nearest whole pound at the end of the study. See Supplemental Material for exact details of the point allocation scheme for all stimuli.

### Data Analysis

Data analysis used R Studio version 2022.7.1.554 (RStudio Team, 2022) and the tidyr (Wickham, 2021), dplyr (Wickham et al., 2021), ggplot2 (Wickham, 2016), effsize (Torchiano, 2020) and Rmisc (Hope, 2013) packages. For the correlations with the questionnaires, exploratory Bayes Factors were calculated with Jamovi Version 2.3.18.0 (The Jamovi Project, 2022) using default settings and default priors, specifically a beta prior with a width of 1. These are reported as *BF*_0+_, which represents the evidence in favour of the null hypothesis (i.e. no correlation) and *BF*_10_, which represents the evidence in favour of the alternative hypothesis over the null.

For the Phase 3 single-stimulus comparisons, the two equivalent Reinforced Controls (C and D) were averaged into a single value: (C + D)/2. In the 2AFC task in Phase 4, participants indicated their preferences using a 100-to-0-to-100 scale with the selected stimulus granted a score of up to +100. For the statistical comparisons, one of the two stimuli was arbitrarily assigned a positive value, and the other a negative value.

To assess the relationship between blocking and conspiracism, a blocking index was calculated. For Phase 3, the blocking index was defined as the difference between the average response to the Reinforced Controls (C and D) and the response to the Described Blocked Stimulus B: ([C + D]/2 − B). For Phase 4, the difference in blocking was assessed by the average preference score for the Described Blocked Stimulus B over the Reinforced Control C in the B versus C comparisons.

## Results

### Phase 1a: Pre-Training

**Figure 2.**
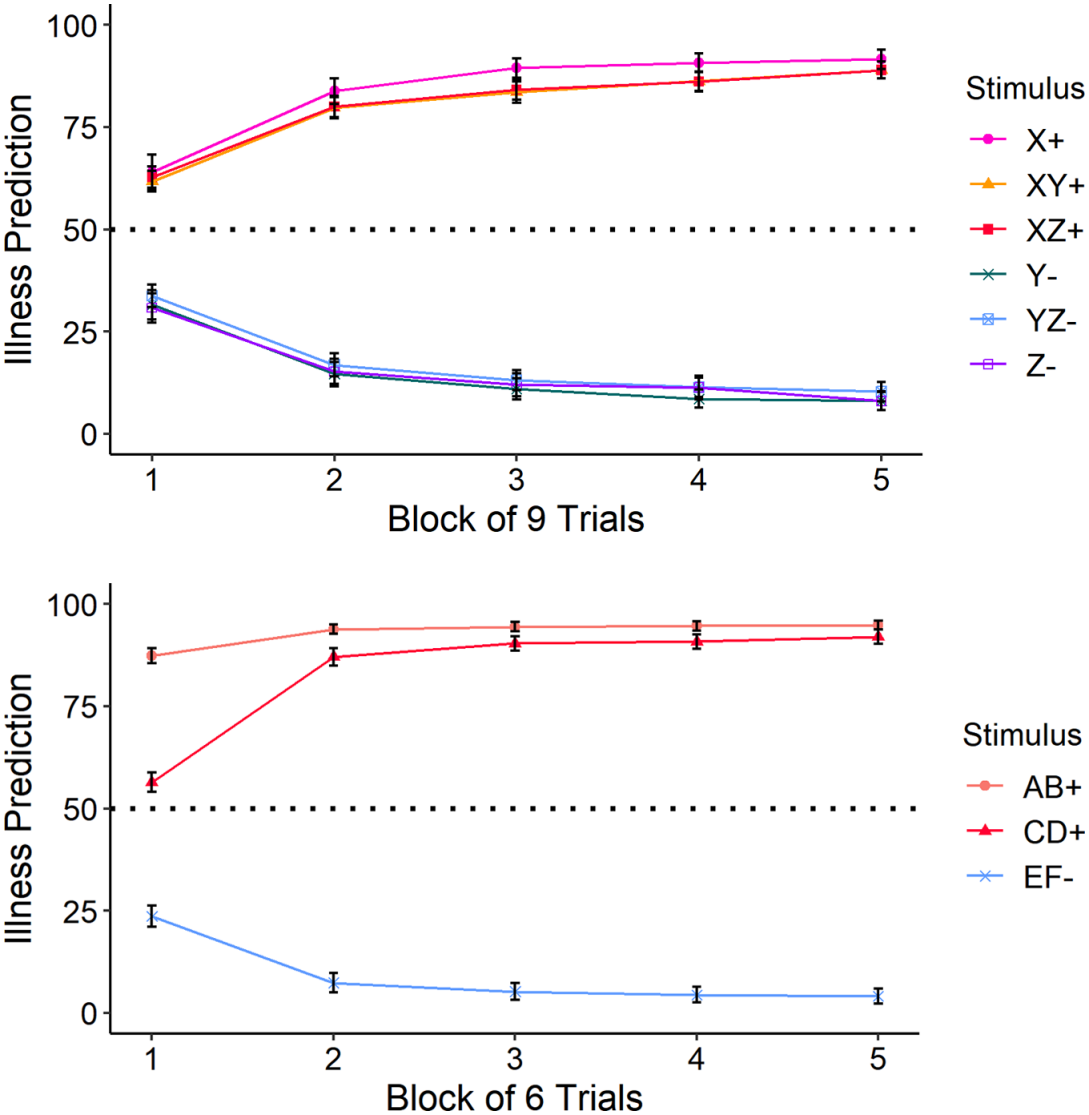
Results from Phases 1a and 2. Participants clearly learned across both phases. Top panel: Mean (±95% CI) illness predictions across all participants by stimulus and block of trials in Phase 1a. Bottom panel: Mean (±95% CI) illness predictions across all participants by stimulus and Block of trials in Phase 2. The dotted lines indicate random or indifferent behaviour.

The top panel of Figure 2 shows that in Phase 1a, participants successfully learnt which locations caused or did not cause illness. Within the first block, ratings were near the midpoint of the scale; by the final block, rewarded and non-rewarded stimuli diverged substantially. Collectively, the Phase 1 data suggest that participants were able to learn about causation in this environment. By the final Block of Phase 1, the participants displayed strong evidence of learning.

### Phase 2: Blocking

The bottom panel of Figure 2 shows a similar pattern. Across the phase, causal and non-causal stimuli diverged. Indeed, participants strongly associated the described blocking compound (AB+) and illness within the first block of Phase 2 trials. This association is evidenced by an average illness prediction of 87.4 ± 2.0 for the first pair of AB+ trials within the first block. A post-hoc, two-tailed *t* test confirmed that participants rated the Described Blocking compound (AB+; *M* = 87.4 ± 2.0) reliably higher than the Reinforced Controls (CD+; *M* = 56.4 ± 2.3; *t*(269) = 19.1, *p* < .001, *d* = 1.61). The high initial rating for the AB+ compound suggests that the description was an effective way of conveying the causal status of the Described Blocking Stimulus (A) and that this transferred effectively to the Described Blocking Compound (AB).

### Phase 3: Single-Stimulus Ratings

**Figure 3.**
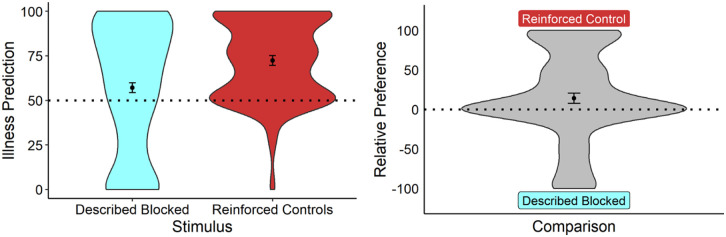
Results from Phase 3 and 4 show clear evidence of blocking. Left panel: Phase 3. Mean (±95% CI) illness predictions across all participants for the Described Blocked Stimulus (B) and Reinforced Control Stimuli (C/D). Right panel: Phase 4. Mean (±95% CI) difference rating across all participants comparing the Described Blocked Stimulus (B), and the Reinforced Control Stimulus (C).

The left panel of Figure 3 depicts the average rating of the key stimuli in Phase 3. Participants showed evidence of blocking compared to a similarly reinforced control. As pre-registered, one-tailed paired *t*-tests confirm that participants rated B, the Described Blocked Stimulus (*M* = 57.1 ± 3.2), significantly lower than C and D, the Reinforced Controls (*M* = 72.3 ± 2.5; *t*(269) = 7.72, *p* < .001, *d* = 0.49). See Table S1 for the full results for all stimuli from this phase.

### Phase 4: 2AFC

The right panel in Figure 3 shows how, in Phase 4, participants selected the Control stimuli over the Blocked Stimulus. This selection reflects a blocking effect whereby participants deemed the Reinforced Control C as 14.1 ± 6.6 points relatively more likely to cause illness than the Described Blocked Stimulus B on the 100-to-0-to-100 scale. This observation was confirmed by a one-sample test against zero, confirming that participants rated the Described Blocked Stimulus as significantly less likely to cause illness than the Reinforced Controls, *t*(269) = 4.23, *p* < .001, *d* = 0.26. The Phase 4 data broadly parallel both the Phase 3 results of this study and the Phase 4 results of previous studies (Kelly & Ludvig, 2025) and provide strong support for the Conspiracy Blocking hypotheses. See Table S2 of the Supplemental Material for the full results from this phase.

### Questionnaires

The two key questionnaire measures participants completed were the General Conspiracy Belief Scale (GCBS) and Belief in Conspiracy Theories Index (BCTI). Both measures have a midpoint of 3.5 with higher ratings indicating higher conspiracy belief. For the 231 participants who completed all questions in the GCBS, the average rating was 2.8 ± 0.1, and the average rating of the 212 participants who completed the BCTI was 2.4 ± 0.1.

**Figure 4.**
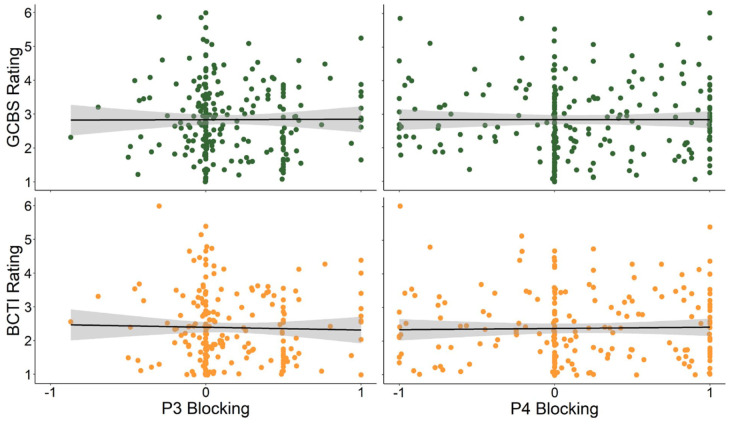
Scatter plots of responses on measures of conspiracism versus strength of blocking in Phase 3 (left) and Phase 4 (right). The top row compares ratings on the General Conspiracy Belief Scale (GCBS) against blocking. The bottom row compares ratings on the Belief in Conspiracy Theories Inventory (BCTI) against blocking. There were no discernible relationships.

Figure 4 shows a scatterplot of the blocking index measure (see above) for Phases 3 and Phase 4 against scores from both the GCBS and BCTI. A one-tailed Pearson’s *r* showed there was no relationship between the GCBS and the Phase 3 blocking index, *r*(226) = .06, *p* = .47, *BF*_0+_ = 11.5. There was also no relationship between the Phase 3 blocking index and the BCTI, *r*(208) = −.03, *p* = .65, *BF*_0+_ = 15.2. Similarly, there was no relationship between the GCBS and the Phase 4 blocking index, *r*(226) = .00, *p* = .50, *BF*_0+_ = 12.1, and no reliable relationship between the Phase 4 blocking index and the BCTI, *r*(208) = .02, *p* = .39, *BF*_0+_ = 9.0. These data provide evidence against the preregistered Conspiracism Correlation hypothesis, suggesting that conspiracy belief does not correlate with the strength of blocking in the learning task.

#### Exploratory Analysis

**Figure 5.**
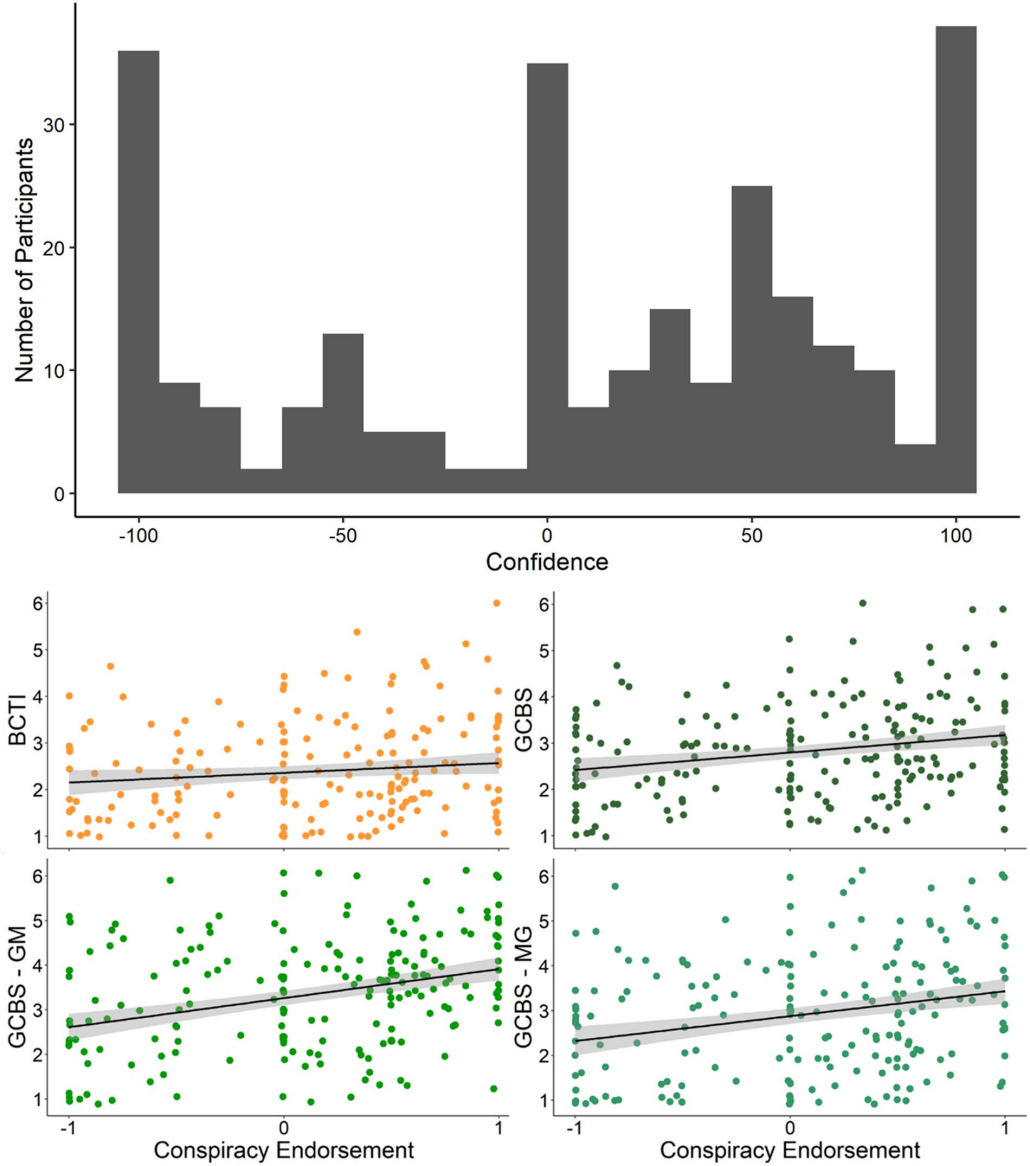
Top-panel: Histogram depicting participant responses to the conspiracy endorsement question: “Did the Benin Government poison Mr Godo?” Mid and bottom panels, scatter plots of average response on the measures of conspiracism versus the extent to which participants endorsed the Benin Government conspiracy (Conspiracy Endorsement). Mid-left panel: Average rating on the BCTI versus Conspiracy Endorsement. Mid-right: Average rating on the GCBS versus Conspiracy Endorsement. Bottom-left: Average response on the GCBS Government Malfeasance subscale versus Conspiracy Endorsement. Bottom-right: Average response on the GCBS Malign Global subscale versus Conspiracy Endorsement. Linear model provides the line of best fit; standard error is indicated. *Note*. BCTI = Belief in Conspiracy Theories Inventory; GCBS = General Conspiracy Belief Scale.

At the conclusion of the experiment, 269 participants responded to the question “Did the Benin Government poison Mr Godo” using a 100-to-0-to-100 scale. Low (negative) scores indicated participants did not think that the Benin government poisoned Mr Godo. High scores indicated they did. The top panel of Figure 5 shows participants weakly endorsed this specific conspiracy (*M* = 9.8 ± 8.1). A post-hoc two-tailed *t*-test against zero confirmed this difference was significant *t*(268) = 2.39, *p* = .02, *d* = 0.15.

Whilst many participants endorsed the Benin conspiracy, this endorsement may not translate into a general propensity to endorse conspiracy theories. This possibility was investigated by comparing participants’ response to the conspiracy-endorsement question and their general conspiracy beliefs. A two-tailed Pearson’s *r* suggested a weak relationship between the conspiracy-endorsement question and the BCTI, *r*(208) = .14, *p* = .04, *BF*_10_ = 0.6, and a stronger relationship with the GCBS, *r*(226) = .25, *p* < .001, *BF*_10_ = 98.4. This relationship was primarily driven by the Government Malfeasance, *r*(226) = .35, *p* < .001, *BF*_10_ = 147,067 and Malign Global subscales, *r*(226) = .28, *p* < .001, *BF*_10_ = 806 (mid and bottom-panels Figure 5). The remaining subscales had correlations with *p* values between .08 and .06. This pattern implies that endorsement of the Benin conspiracy in the learning task was linked to general conspiracism as measured by the GCBS.

The supplement details additional exploratory correlations suggesting no relationship between the blocking index employed above and the measures of political orientation (see Figure S2).

## Discussion

This experiment adapted a classic blocking paradigm to investigate the role of associative learning in the learning and maintenance of conspiracy beliefs (Dickinson et al., 1984; Kamin, 1969). Participants were told of the location of a possible conspiracy via an explicit description. This description prompted strong learning of the compound stimulus in which that location was present and blocked the association with illness for the second location from that compound stimulus. Participants on aggregate endorsed a conspiracy implied by what they had learnt, extending existing work on novel conspiracy beliefs (Bost & Prunier, 2013; Imhoff & Lamberty, 2017; van Prooijen & van Dijk, 2014). There was, however, no evidence that those who endorse conspiracy theories in general were more susceptible to blocking, even with a conspiratorial framing.

This study represents one of the first investigations into the processes which determine the learning and maintenance of conspiracy beliefs. In this conspiratorial context, the classic behaviour of blocking emerged. As such, the present findings provide a step towards understanding conspiracy belief more broadly. In general, most studies into conspiracy belief are correlational, with experimental studies being less common (Orosz et al., 2016; Sassenberg et al., 2023; Van Der Linden et al., 2023). As noted, previous research has investigated various biases linked to conspiracism, including jumping to conclusions (Hattersley et al., 2022; Moulding et al., 2016; Pytlik et al., 2020), intuitive thinking (Hattersley et al., 2022; Swami et al., 2014; van Prooijen, 2016) and confirmation bias (Quattrociocchi et al., 2016). The present study adds a key element, examining how this information is acquired and maintained.

To our knowledge, this is the first attempt to use an associative learning methodology to investigate conspiratorial belief. Beyond blocking, other approaches from associative learning have been used to investigate social psychological phenomena. For instance, contingency learning tasks have been used to investigate the illusion of causality in believers in the paranormal or how left- or right-wing voters assess the success of government policies (Blanco et al., 2015, 2018). Here, participants learnt a novel conspiracy-congruent scenario, which blocked learning about an alternative cause; participants also endorsed a conspiracy based off that scenario as having occurred (see Figures 3 and 5). If associative methodologies can capture the acquisition and maintenance of conspiratorial beliefs, the long history of associative learning could be leveraged to better understand how these beliefs are learnt, how their transmission could be disrupted, and how their sometimes-baseless conclusions can be corrected. This possibility is important given the negative consequences of belief in conspiracy theories (Jolley & Douglas, 2014a, 2014b; Lewandowsky et al., 2013; van der Linden, 2015).

The present pattern of blocking resembles previous work using described outcomes with abstract shapes in a non-conspiratorial paradigm (Kelly & Ludvig, 2025). This similarity both suggests that the processes underlying the behaviour observed generalise beyond the specifics of the present framing and the robustness of the blocking presently observed. By extension, conspiracy beliefs may be underpinned by broad learning processes like many other abstract beliefs (Blanco et al., 2015, 2018; Skinner, 1948).

Within the associative learning literature, there are competing explanations for the expression of cue-competition effects like blocking. In animals, blocking is often assumed to be an automatic, bottom-up consequence of stimulus-reward pairings (Mackintosh, 1975; Pearce & Hall, 1980, Rescorla & Wagner, 1972; but see Beckers et al., 2006). In humans, however, there is substantial evidence that blocking effects may follow from deliberative, top-down, reason-led processes (De Houwer et al., 2002; Lovibond et al., 2003, Schmidt & De Houwer, 2019). This research has led to claims that blocking in humans exclusively results from formalised propositions of the form “I know this (blocking) stimulus is causal, I know this (blocked) stimulus is not causally necessary, therefore this (blocked) stimulus is non-causal” (Mitchell et al., 2009). Even for humans, however, whether blocking necessarily results from top-down reason-based processes is still contested (Livesey et al., 2019; see Kelly & Ludvig, 2025 for a more extensive discussion).

This paper offers a clearer potential mechanism for the maintenance of an already-learned conspiracy belief than it does for how a conspiracy is initially learnt. The study does, however, still contribute to the questions of learning for several reasons. First, the possible conspiracy that participants encounter is novel and fictitious. For the conspiracy to have any impact on behaviour (i.e. blocking), this impact must result from learning which has occurred during the study. Second, participants were introduced to a *possible* conspiracy. Participants had to take an additional step to endorse this as an actual conspiracy (Figure 5). Indeed, participants took this step despite their environment offering an alternative possible explanation for what was encountered (i.e. the blocked stimulus). That said, further investigation of the mechanics of initial learning as well as maintenance could prove fruitful for future research.

In this study, there was, however, no relationship between blocking and conspiracy belief in general (see Figure 5). One possible explanation is that the task may have lacked the ecological validity required for a relationship between blocking and conspiracism to emerge. The positive correlations between endorsement of the specific Benin conspiracy and the BCTI and GCBS suggest this is unlikely to be the case (see Figure S2). Bayesian analyses suggested there was a relationship between the GCBS as a measure of conspiracy belief and endorsement of the possible Benin conspiracy, although there was no equivalent relationship between the BCTI and the possible Benin conspiracy. This difference may reflect a dissimilarity in how the measures are constructed. The BCTI is a list of specific conspiracy theories to which participants choose their level of endorsement, whereas the GCBS offers more general statements that identify a conspiratorial mentality. Whilst these two kinds of measures are related, they do not always produce equivalent results (Imhoff, 2024; Imhoff et al., 2022; Sutton & Douglas, 2020; Sutton et al., 2024; Trella et al., 2024). The present results may even reflect a general tendency for individuals who believe in conspiracy beliefs to adopt new conspiracy beliefs, forming a self-sustaining, monological network of conspiracy beliefs (c.f. Goertzel, 1994; Wood et al., 2012). Regardless, in broad terms, there is evidence of the task’s ecological validity.

Another explanation for the lack of a link between the measures of conspiracy belief and blocking is that knowledge of a conspiracy theory is not sufficient to become a conspiracy theorist. This information presumably must be retained and revisited for it to influence behaviour. This is impossible without learning and initial maintenance. These latter steps, however, may be where those who endorse items on the BCTI or the GCBS differ from those who do not. Alternatively, as previously noted, those who endorse conspiracy theories may learn and maintain beliefs similarly to those who do not. The difference in beliefs may result from the environments in which individuals learn, or indeed unlearn (Costello et al., 2024).

This study explicitly sought to provide insight into how participants respond to a novel conspiracy theory prior to encountering an alternative explanation. This approach clearly maps onto some aspects of the generation and maintenance of conspiracy beliefs better than others. Exposure to a novel conspiracy belief is possibly analogous to either people researching something unknown or trying to understand a “breaking news” event where there is a lack of clarity on the causes of an event. There are alternative situations in which one may encounter a conspiracy theory, such as dissemination across social media, and it may be in these situations where conspiracy believers display unusual behaviour (Del Vicario et al., 2016).

The experimental paradigm presented in this paper provides a flexible framework to investigate the learning and maintenance of conspiratorial beliefs. For example, the protocol could be amended to investigate backward blocking (Shanks, 1985). In the current version, participants were first exposed to the conspiracy-congruent blocking stimulus (Phase 1b) then exposed to the blocking-blocked stimulus in compound (Phase 2). This order could easily be reversed, potentially providing insights into how people incorporate conspiratorial-congruent information into already-learned behaviours. Alternatively, a question like the one asked following Phase 4 could be inserted earlier into the task (i.e. “Did the Benin Government poison Mr Godo?”). Conspiracy believers presented with such an explicit opportunity/prompt to interpret what they had learnt as an actual conspiracy may react differently to non-believers, potentially leading to different blocking behaviours.

In conclusion, this experiment suggests that associative learning can be used to gain insight into the learning, maintenance and endorsement of conspiracy beliefs. Participants were told about a novel conspiracy-congruent story, which blocked future learning about alternate causes. Moreover, participants endorsed a specific conspiracy theory implied by the story despite having an alternative causal explanation available. There was, however, a lack of clear evidence linking blocking in this task to conspiracism in general. Given the possibly self-sustaining nature of conspiracy beliefs, this initial step in understanding how conspiracy beliefs are acquired and maintained could help inform how to lessen endorsement of them and, by extension, lessen the detrimental impact these beliefs have on us all.

## Supplemental Material

sj-docx-1-qjp-10.1177_17470218251396472 – Supplemental material for Associative Learning in a Conspiratorial Frame Supplemental material, sj-docx-1-qjp-10.1177_17470218251396472 for Associative Learning in a Conspiratorial Frame by Tom Kelly, Michael Hattersley and Elliot A. Ludvig in Quarterly Journal of Experimental Psychology

## Notes

1. The preregistration details two additional hypotheses and pre-registered analyses, which are reported in the Supplemental Material. The primary hypotheses had slightly different names in the pre-registration, which have been updated for clarity.
