# Supplementary material for: Associative Learning in a Conspiratorial Frame
Source: Q J Exp Psychol (Hove). 2025 Nov 1;79(8):1985–98. doi: 10.1177/17470218251396472 (PMC13400819; doi:10.1177/17470218251396472)
Supplement: sj-docx-1-qjp-10.1177_17470218251396472 – Supplemental material for Associative Learning in a Conspiratorial Frame [file sj-docx-1-qjp-10.1177_17470218251396472.docx]

# Supplementary Materials for:

#

# **Associative Learning in a Conspiratorial Frame**

Tom Kelly^1,2^, Michael Hattersley^3^, Elliot A. Ludvig^1^

^1^ University of Warwick

Department of Psychology

Coventry

UK

^2^ University College Dublin

Sutherland School of Law

Dublin 4

Ireland

^3^ Slovak Academy of Sciences

Institute of Experimental Psychology

Bratislava, Bratislava Region 814 38

Slovakia

Corresponding Author: Tom Kelly

Department of Psychology,

University of Warwick,

Coventry, CV4 7AL,

United Kingdom

thomas.m.kelly@warwick.ac.uk

https://orcid.org/0000-0001-8444-1767

## **Incentive structure**

In Phases 1, 2 and 3, participants were asked to indicate the likelihood that their patient/Mr Godo was ill using a 0-to-100 scale. Points were allocated as follows: Participants received bonus points when they correctly stated that a stimulus caused illness, and they lost points for incorrectly indicating that a stimulus caused illness. Participants received points equivalent to the score on the scale, such that, for example, rating a stimulus that caused illness at 97 scored 97 points and rating a stimulus that did not cause illness at 43 lost 43 points. They could win up to 100 points for stimuli that caused illness and lose up to 100 for those that did not.

In Phase 3 with single stimuli, participants still gained or lost points as above for stimuli that clearly caused or did not cause illness. For the blocking or control stimuli, for which participants could not know the predictive value of with certainty, they neither gained nor lost points (Stimuli B, C, D, & N). This evaluation was based on the non-additive causal structure of the experiment. Two predictive stimuli in compound provided the same outcome as one. If participants could infer based off this structure that a stimulus was predictive or not, they could gain or lose points as they did for judging the compounds in Phase 1a or 2.

In Phase 4, participants gained points equivalent to their rating on the 100-to-0-to-100 scale if they picked the dominant option when one was available. They, however, lost points equivalent to their rating if they failed to pick a dominant option when available. Specifically, participants were rewarded for picking A or punished for picking E over any of the alternatives. Participants gained points for choosing C over any stimulus except A. If neither option was dominant (e.g., B vs N), participants received 100 points for selecting the middle of the 100-to-0-to-100 scale, otherwise they earned 100 – 2*(slider rating). This scoring system meant participants earned points for being close to 0 but lost up to 100 by selecting an extreme value.

Participants earned £1 for each 3000 bonus points earned. There was a maximum of 9000 bonus points available in the experiment (which would provide a £3 bonus). The bonus was rounded up to the nearest whole pound at the end of the study.

## **Stimulus Materials**

The following images were used as location stimuli:

| Stimulus Image | Stimulus Label | Stimulus Image | Stimulus Label |
| --- | --- | --- | --- |
| 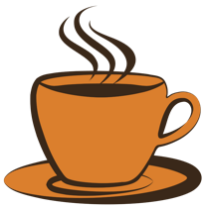 | Coffee shop | 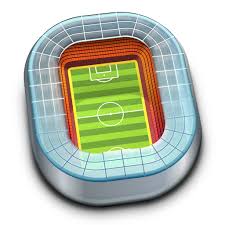 | Local stadium |
| 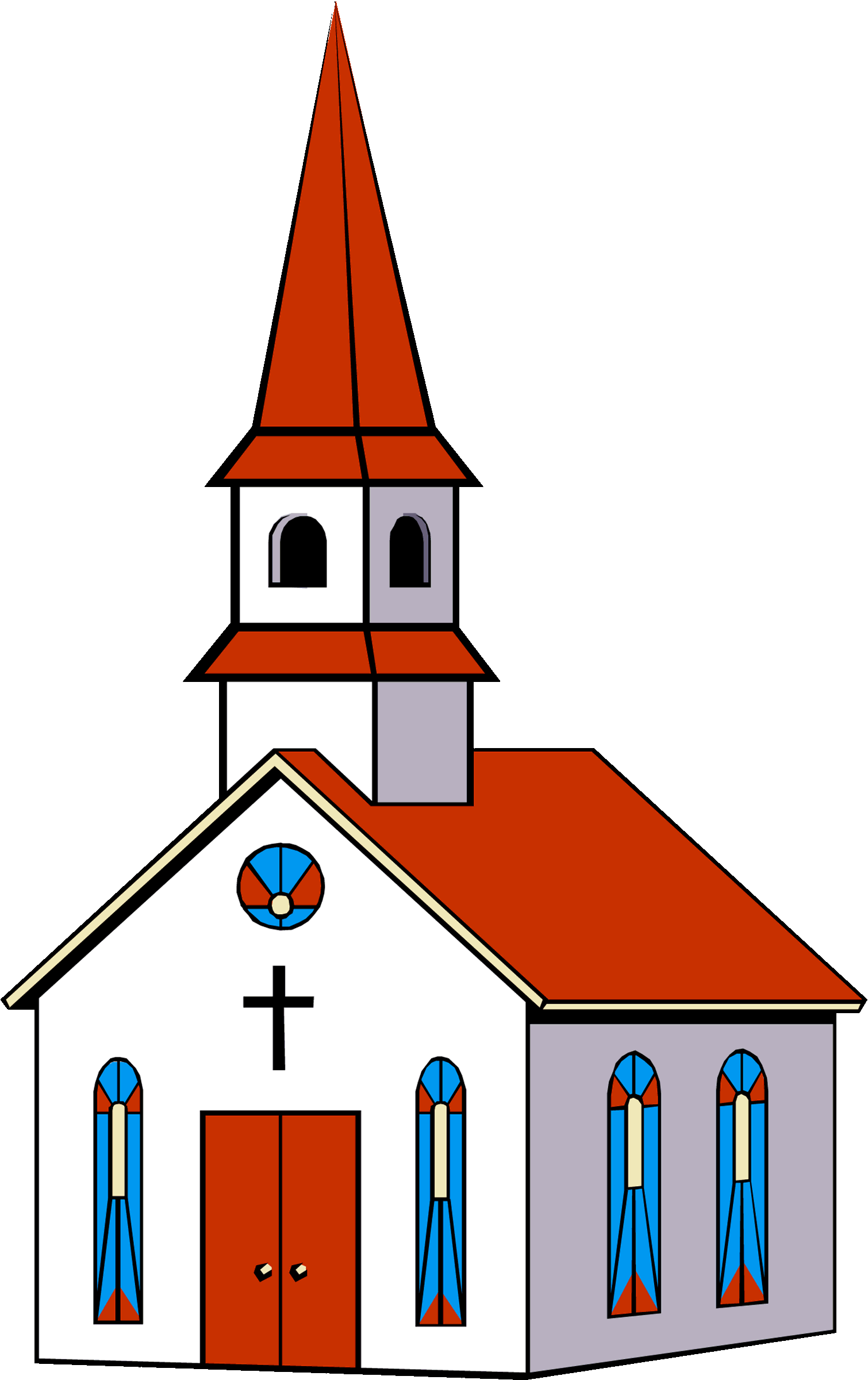 | Church | 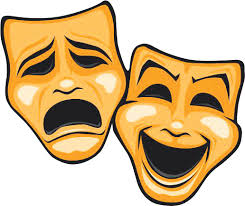 | National Theatre |
| 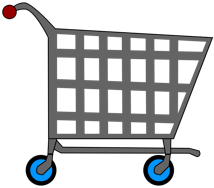 | The supermarket | 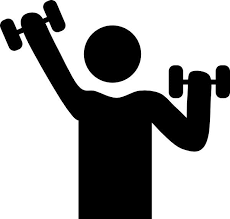 | Gym |
| 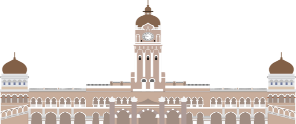 | Parliamentary Office | 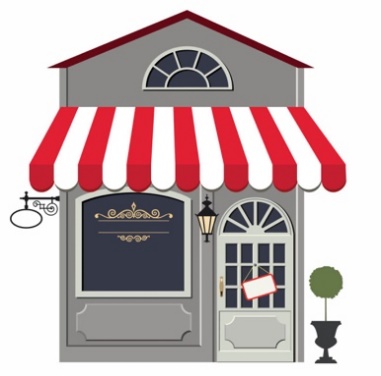 | Favourite restaurant |
| 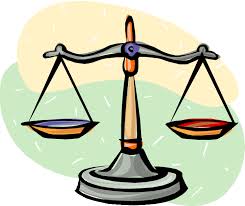 | Lawyer's practice | 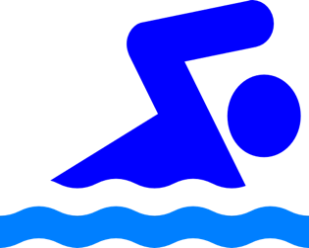 | Swimming baths |

## Belief in Conspiracy Theories Inventory (BCTI) - Swami et al., 2010, 2011

| No. | Question |
| --- | --- |
| 1. | A powerful and secretive group, known as the New World Order, are planning to eventually rule the world through an autonomous world government, which would replace sovereign government. |
| 2. | SARS (Severe Acute Respiratory Syndrome) was produced under laboratory conditions as a biological weapon. |
| 3. | The US government had foreknowledge about the Japanese attack on Pearl Harbour, but allowed the attack to take place so as to be able to enter the Second World War. |
| 4. | US agencies intentionally created the AIDS epidemic and administered it to black and gay men in the 1970s. |
| 5. | The assassination of Martin Luther King, Jr. was the result of an organised conspiracy by US government agencies such as the CIA and FBI. |
| 6. | The Apollo moon landings never happened and were staged in a Hollywood film studio. |
| 7. | Area 51 in Nevada, US, is a secretive military base that contains hidden alien spacecraft and/or alien bodies. |
| 8. | The US government allowed the 9/11 attacks to take place so that it would have an excuse to achieve foreign (e.g., wars in Afghanistan and Iraq) and domestic (e.g., attacks on civil liberties) goals that had been determined prior to the attacks. |
| 9. | The assassination of John F. Kennedy was not committed by the lone gunman, Lee Harvey Oswald, but was rather a detailed, organised conspiracy to kill the president. |
| 10. | In July 1947, the US military recovered the wreckage of an alien craft from Roswell, New Mexico, and covered up the fact. |
| 11. | Princess Diana's death was not an accident, but rather an organised assassination by members of the British royal family who disliked her. |
| 12. | The Oklahoma City bombers, Timothy McVeigh and Terry Nichols, did not act alone, but rather received assistance from neo-Nazi groups. |
| 13. | The Coca Cola company intentionally changed to an inferior formula with the intent of driving up demand for their classic product, later reintroducing it for their financial gain. |
| 14. | Special interest groups are suppressing, or have suppressed in the past, technologies that could provide energy at reduced cost or reduced pollution output. |
| 15. | Government agencies in the UK are involved in the distribution of illegal drugs to ethnic minorities. |

*Note:* Participants were instructed: “Please consider the following statements and indicate your agreement with each in turn from 1 (Strongly Disagree) to 6 (Strongly Agree).”

## Generic Conspiracist Belief Scale (GCBS) - Brotherton, French and Pickering 2013

| No. | Question | Subscale |
| --- | --- | --- |
| 1. | The government is involved in the murder of innocent citizens and/or well-known public figures and keeps this a secret | Government malfeasance |
| 2. | The government permits or perpetrates acts of terrorism on its own soil, disguising its involvement | Government malfeasance |
| 3. | The government uses people as patsies to hide its involvement in criminal activity | Government malfeasance |
| 4. | The power held by heads of state is second to that of small unknown groups who really control world politics | Malevolent global conspiracies |
| 5. | A small, secret group of people is responsible for making all major world decisions, such as going to war | Malevolent global conspiracies |
| 6. | Certain significant events have been the result of the activity of a small group who secretly manipulate world events | Malevolent global conspiracies |
| 7. | Secret organisations communicate with extra-terrestrial but keep this fact from the public | Extra-terrestrial cover-up |
| 8. | Evidence of alien contact is being concealed from the public | Extra-terrestrial cover-up |
| 9. | Some UFO sightings and rumours are planned or staged in order to distract the public from real alien contact | Extra-terrestrial cover-up |
| 10. | The spread of certain viruses and/or diseases is the result of the deliberate, concealed efforts of some organisation | Personal well-being |
| 11. | Technology with mind-control capacities is used on people without their knowledge | Personal well-being |
| 12. | Experiments involving new drugs or technologies are routinely carried out on the public without their knowledge or consent | Personal well-being |
| 13. | Groups of scientists manipulate, fabricate, or suppress evidence in order to deceive the public | Control of information |
| 14. | New and advanced technology which would harm the current industry is being suppressed | Control of information |
| 15. | A lot of important information is deliberately concealed from the public out of self-interest | Control of information |

*Note:* Participants were instructed: “Please consider the following statements and indicate your agreement with each in turn from 1 (Strongly Disagree) to 6 (Strongly Agree).” This measure also included an attention check “This question is an attention check. Please select the number "five".”

## Social and Economic Conservatism Scale (SECS) - Everett 2013

“Please indicate the extent to which you feel positive or negative towards each issue. Scores of 0 indicate greater negativity, and scores of 100 indicate greater positivity. Scores of 50 indicate that you feel neutral about the issue.”

1. Abortion (reverse scored). (S)
2. Limited government. (E)
3. Military and national security. (S)
4. Religion. (S)
5. Welfare benefits (reverse scored). (E)
6. Gun ownership. (E)
7. Traditional marriage. (S)
8. Traditional values. (S)
9. Fiscal responsibility. (E)
10. Business. (E)
11. The family unit. (S)
12. Patriotism. (S)

## Self-Rated Left-Right wing preference


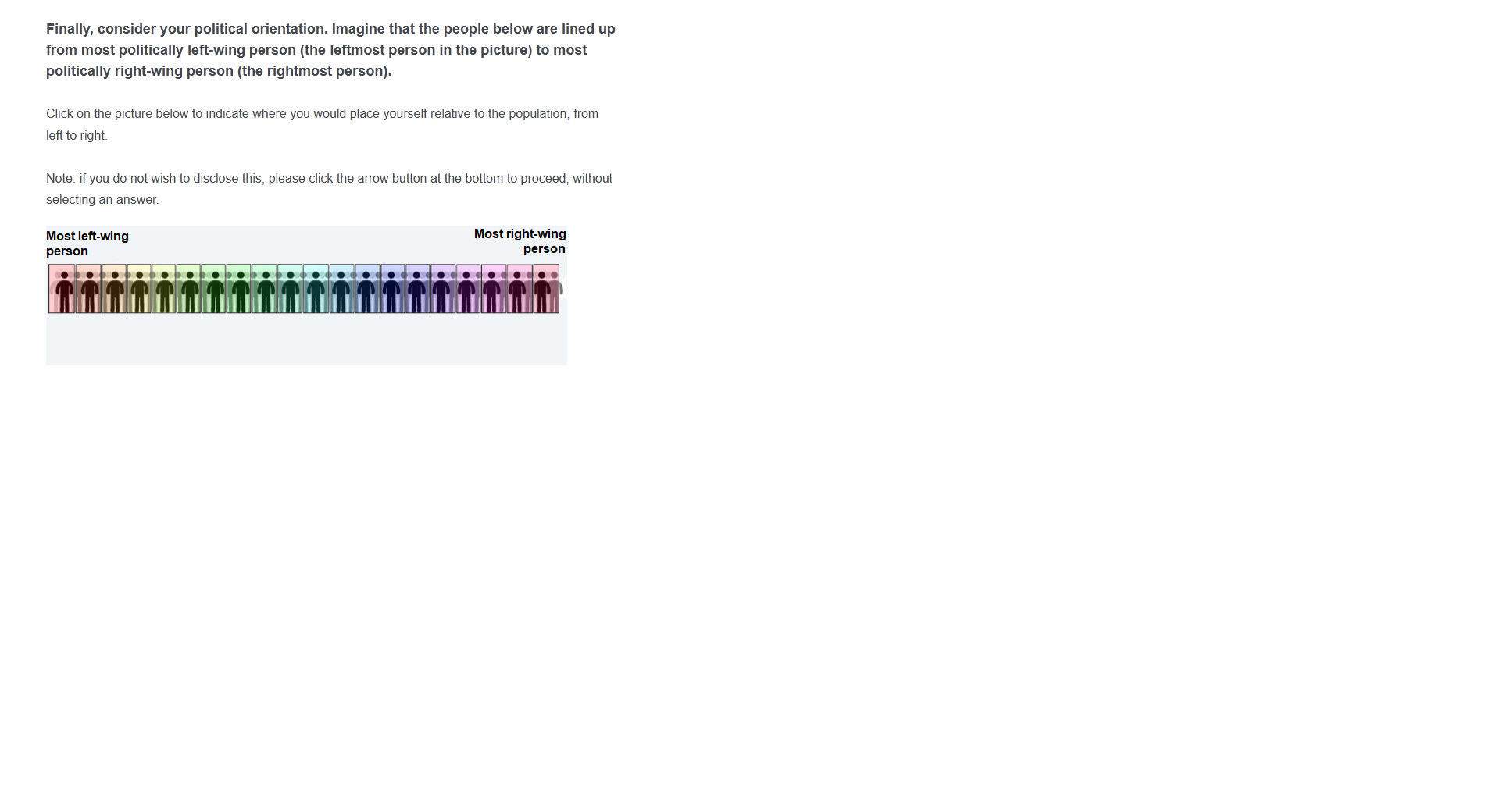


## **Conspiracy Description**

In Phase 1b (Described stimulus), Participants were introduced to the story of their patient “Mr Godo”. The story of Mr Godo was inspired from the conspiracy theory literature, specifically van Prooijen & van Dijk (2014). Mr Godo was introduced as follows:

“Today your patient is Mr Yayi Godo. Mr Godo is a prominent opposition politician in the West African country of Benin. His anti-crime, anti-corruption platform has gained significant media attention. Recently polls suggested he would make a more popular presidential candidate than the more established and current front runner for the presidency Ms Murielle Deguenon.

His anti-corruption drive has upset many within his party and even more outside it.

Mr Godo has always suffered from allergies, but recently they have become debilitating. You ask Mr Godo to keep a diary of where he spends most of his time in the day and how he felt afterwards.”

Participants were able to advance from this page after 7 s. This pause was then be followed by reports suggesting a specific location is the cause of his illness:

“News of Mr Godo’s worsening illness eventually becomes public. National newspaper The Porto-Novo Record reports that Mr Godo’s illness is due to deliberate exposure to industrial cleaning products banned in Benin but available in neighbouring countries.

They allege Mr Godo’s Gym is where he’s been exposed to these products.

They claim that the Gym is owned by a major donor of the government and is actively managed by a member of the ruling party. They report that the entire cleaning staff had recently been laid off without notice and replaced without any explanation.”

Note that the precise wording of this message changed depending on how the stimuli were initially randomised.

**Feedback**

Feedback text and Images for Phases 1a and 2:

| Feedback Text | Feedback Image |
| --- | --- |
| P1a “The Patient was ill”  P2 “Mr Godo was ill” | 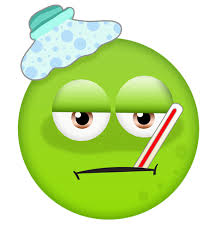 |
| P1a “The Patient was not ill”  P2 “Mr Godo was not ill” | 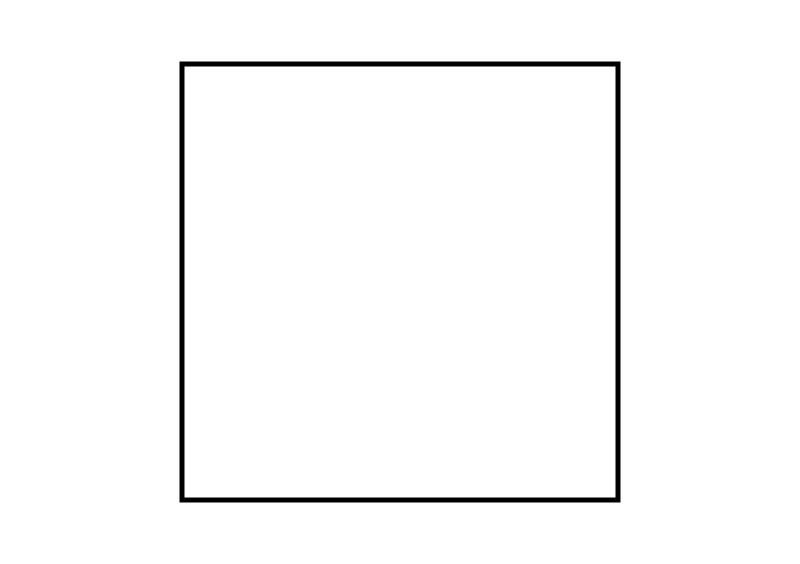 |

## **Instructions**

*Welcome screen:*

"Welcome to the experiment. In this study you are taking the role of an allergist. Allergists help diagnose people’s allergies – they help people understand what it is in their environment that is making them ill. People can have allergic responses to things that they eat, things that they inhale, or things that they touch. We’re interested in how people learn which places might cause illness. We’ll show you one or two locations where your patients spent a lot of time during the day and then we’ll show you if the patient felt ill that evening."

*Instructions screen:*

"In Parts 1, 2 and 3 of this experiment you'll be asked to rate how likely your patient will be ill. You will be asked to rate your confidence on a 0 to 100 scale. Accuracy scores 'bonus points'. Pick the location on the scale that best reflects your belief to score the most points. At the end of Parts 1 and 2 you'll see how many bonus points you have earned thus far. Your task is the same in every round. Each round is part of the same game."

*Scale Instructions:* "Each location or location pair may be worth up to 100 points, Locations that cause illness can gain you up to 100 points. Locations that do not cause illness can lose you up to 100 points, 30 points are worth 1p at the end of the experiment."

*Feedback Screen:*“The following Images are your feedback. Use them to learn if a location causes illness. Press Space to continue.”

*Phase 2 introduction:*

"Thank you for completing Part 1, In Part 2 you will again be asked to state if your patient will be ill. You are still using the same scale to predict likelihood."

*Phase 3 introduction:*

"Thank you for completing Part 2, In Part 3 you will again be asked to state if Mr Godo will be ill. You are still using the same scale to predict likelihood."

*Phase 4 Introduction:*

"Thank you for completing Part 3, In Part 4 you will be asked to choose which location is more likely to make Mr Godo ill. You are choosing between locations You are picking the location that is more likely to cause illness."

## **Supplemental Analyses**

The pre-registrations for this Experiment (https://osf.io/m3ywf/) detailed additional hypotheses omitted from the main manuscript for clarity and simplicity.

### **Blocking relative to Novel Controls^[[1]](#footnote-1)^**

The pre-registration detailed an analyses comparing the Blocked Stimulus to a Novel Control: In Phase 3, additional paired one-tailed t-tests confirmed that participants rated the Described Blocked Stimulus and the Reinforced Controls significantly higher than the Novel Control (*M* = 31.3±3.3); *t*(269) = 10.41, *p* < 0.001, *d* = 0.78 and *t*(269) = 20.30, *p* < 0.001, *d* = 1.58 respectively. In Phase 4, there was no evidence for blocking relative to a Novel Control (N); For the B vs N comparison participants preferred stimulus B with an average rating of (*M* = -35.8±6.2). This observation was confirmed by a one-sample test against zero where the Described Blocked Stimulus was deemed as significantly more likely to cause illness than the Novel Control; *t*(269) = 11.4, *p* < .001, *d* = 0.69.

### **Supplementary Control Analysis**

An additional Hypothesis was pre-registered: Probability Sensitivity. The hypothesis predicts that the reinforced controls should be higher than the novel controls because of their relative reinforcement rate, even though both are causally ambiguous. In Phase 3, participants rated the Reinforced Controls C/D (*M* = 72.32±2.5) significantly higher than the Novel Control N (31.3±3.3); *t*(269) = 20.30, *p* < 0.001, *d* = 1.58. In Phase 4, participants’ average response for stimulus N on the on the N vs C comparison was (*M* = 46.8±5.5) in favour of C. Participants rated the Reinforced Controls as significantly more likely to cause illness than the Novel Control; *t*(269) = 17.79, *p* < 0.001, *d* = 1.08. These results provide evidence that people were sensitive to the probability of a stimuli being associated with illness in this task.

- - 1. **Exploratory Questionnaire Analysis**

In addition to the conspiracy questionnaire measures participants completed the Social and Economic Conservatism Scale (0-to-100 scale) and a self-rated left/right measure of political preference (1-to-20 scale). 238 participants completed all items of the economic measures in the SESC (Everette, 2013; *M* = 57.5±1.8), and 248 completed all items of the social measures of the same scale (*M* = 58.8±1.4). The SESC has a midpoint of 50 with higher ratings indicating higher conservatism. 265 respondents indicated their position on the left/right scale (*M* = 9.4±0.6). The self-rating had a midpoint of 10.5 with higher ratings indicating higher conservatism. There was a small number of participants (4 for the social, 4 for the economic subscales of the SECS and 4 for left/right scale) who completed all measures within one or more of the questionnaires despite having quit, crashed, or otherwise prematurely terminated their participation in the allergy-detection task.

Figure S1 shows a scatterplot of the blocking index measure (see main manuscript) for Phases 3 and Phase 4 against the social and economic ratings of the SECS and the self-rated left right scale. A two-tailed Pearson’s r showed there was no relationship between the social, *r*(242) = -.004, *p* = . .95, or the economic, *r*(232) = .03, *p* = .63, subscales of the SESC scale and the Phase 3 blocking index. Additionally, there was no relationship between the self-rated scale and Phase 3 blocking; *r*(259) = -.05, *p* = .38. Similarly a two-tailed Pearson’s r showed there was no relationship between the social, *r*(242) = -.05, *p* = .41, or the economic, *r*(232) = .02, *p* = .72, subscales of the SESC scale and the Phase 4 blocking index, nor was there any relationship between the self-rated scale and Phase 4 blocking; *r*(259) = -.07, *p* = .20.


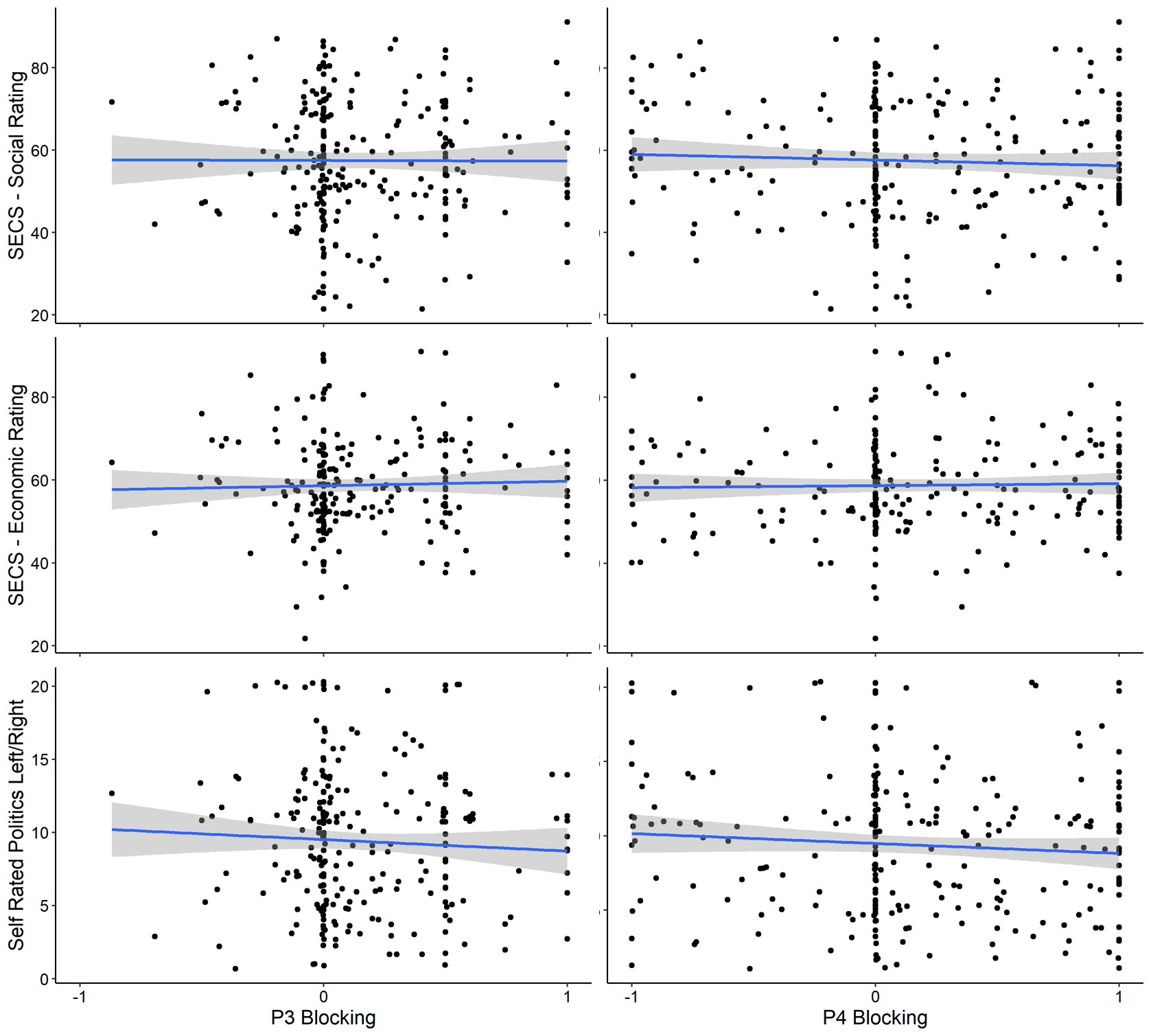


Figure S.1 *Scatter plots of participants’ political views vs the extent to which they displayed blocking in Phases 3 and 4. The left column corresponds to Phase 3 blocking, and the right column to Phase 4 blocking. The top row represents the social rating subscale from the Social and Economic Conservatism Scale (SECS). The middle row represents the economic rating on the SECS. The bottom Row represents participants’ self-rated left/right rank. Linear model provides the line of best fit; standard error indicated by shaded area.*

## **Full results Phase 3**

Table S1. Mean preferences by comparison from the Single-Stimulus Ratings in Phase 3, where higher values indicate a stronger association with illness, and a value of 50 indicates indifference or random behaviour.

| Label | Stimulus | P3 Illness prediction |
| --- | --- | --- |
| Described Blocking Stimulus | A | 92.9 ± 2.2 |
| Described Blocked Stimulus | B | 57.1 ± 3.6 |
| Reinforced Control | C | 71.7 ± 3.4 |
| Reinforced Control | D | 73.0 ± 3.3 |
| P2 Unreinforced | E | 6.0 ± 2.1 |
| P2 Unreinforced | F | 5.8 ± 2.0 |
| Novel Control | N | 31.3 ± 3.2 |
| Training Reinforced | X | N/A |
| Training Unreinforced | Y | N/A |
| Training Unreinforced | Z | N/A |

## **Full results Phase 4**

Table S2. Mean preferences by comparison from the 2AFC task, where higher values indicate a stronger association with illness for Stimulus 1, and a value of 0 indicates indifference or random behaviour.

| Stim 1 | Stim 2 | Av Pref |
| --- | --- | --- |
| A | E | 88.8 ± 3.4 |
| A | N | 77.2 ± 4.4 |
| C | E | 71.5 ± 4.8 |
| B | E | 59.8 ± 5.4 |
| A | B | 55.3 ± 6.0 |
| C | N | 49.8 ± 5.5 |
| A | C | 47.9 ± 6.0 |
| B | N | 35.8 ± 6.2 |
| N | E | 35.5 ± 5.3 |
| C | B | 14.1 ± 6.5 |

*Note. A = Described Blocking Stimulus, B = Described Blocked Stimulus, C = Reinforced Control, E = Phase 2 Stimulus not predictive of illness, N = Novel Control stimulus introduced in Phase 3.*

1. Please note an inconsistency between Hypothesis 1b and the analysis plan for 1b listed in the pre-registration. Reported here is the pre-registered analysis. [↑](#footnote-ref-1)
